# Editorial

**Published:** 1871-08

**Authors:** 


### Editorial.

Preliminary Education.—The Indiana State Medical Society, at its recent Annual Meeting, adopted the following resolution, which ought to be carried into effect by every local medical society in the whole country:

"*Resolved,* That the several county societies in this State be requested to appoint a Board of three competent Members of their body to examine into the general education and intelligence of any one desiring to become a student of medicine, and that such societies permit none of their members to receive a student who does not present a certificate of proper qualification from such Board."

There is no one measure that would do as much to increase the reputation and usefulness of the profession as that which will ensure a fair and liberal general education for such as enter upon the study of medicine.

American Medical Association.—We are informed by the Secretary that he will issue the Proceedings of the recent meeting of the Association at San Francisco, in pamplet form, at 25 cents per copy. All who desire copies can forward the amount with the address, directly, to the Secretary, Wm. B. Atkinson, M.D., 1400 Pine Street, Philadelphia.

American Medical Association.—Having attended the recent meeting of this Association at SanFrancisco, California, and attentively noted all that was said and done during its several sessions, we have been surprised in reading the editorial comments on that meeting in several of our exchanges. They not only characterize the meeting as a failure in a scientific and professional aspect, but they flippantly speak of its doings as disorderly, and even disgraceful. At first we were unable to account for these unfavorable representations; but on examining the matter more closely, we found these disparaging comments exclusively in journals whose editors were not present at the meeting, but who had evidently formed their opinions altogether from the imperfect and erroneous reports of the daily press of SanFrancisco.

Nearly all of them had, indeed, copied the reports of the daily press, with all their errors, into their own columns, and some had gone further, and copied the unfriendly slurs and criticisms found in secular papers that never lose an opportunity to disparage legitimate medicine. By adopting such a course those journalists induce their readers to suppose that the members of the Association who assembled in SanFrancisco, did little else than to wrangle over questions pertaining to *woman's* rights and wrongs, while the better part of the profession resident in SanFrancisco looked on with dignified contempt. We do not hesitate to say that all these severely disparaging comments on the doings of the recent meeting, are exceedingly unjust, both to the residents in SanFrancisco and the Association as a whole. There has not been a meeting of the Association, since its organization, in 1847, during which the business of the general sessions was transacted with more regularity, general decorum, and individual courtesy, than during the one in SanFrancisco.

Indeed, the whole amount of time consumed in the discussions in reference to admitting delegates from hospitals and colleges exclusively for the care and education of females, and consultations with female physicians, was not equal to that consumed in trying to elect officers at the first anniversary meeting in Baltimore.

And yet these discussions are magnified by full reports in true sensational newspaper style, claimed to be verbatim, yet so imperfect as to contain paragraphs altogether devoid of meaning, while many other reports and discussions of much more professional importance, are either passed over in silence, or merely alluded to. In this way an entirely false coloring is given to the whole course of proceedings in the general sessions of the Association. This false coloring is made still more marked by the reporters paying no attention to the doings in the Section Meetings, where all the strictly scientific and practical professional work of the Association is done. It is not our object to find fault with the newspapers or their reporters. They naturally make the most of discussions on such miscellaneous topics as will most readily attract the attention and gratify the curiosity of their readers. To them an hour spent in discussing the professional status of woman is a god-send; while a most valuable essay on the nature and treatment of disease would neither be intelligible or interesting to them or their readers. Hence they will magnify their account of the first into a column, and compress that of the latter into a line.

We have some reason to complain, however, when editors of strictly medical periodicals copy, uncorrected, these imperfect and one-sided newspaper reports, and found on them disparaging and injurious comments concerning the Association. And we shall have still greater cause to complain, if the Secretary in making up his official record for publication, shall copy from the same imperfect source. The truth is, the meeting in San-Francisco was characterized by a fair degree of success in all its interests. The number in attendance was fair; the reception by the local profession of SanFrancisco, embracing all their leading and worthy men, was cordial and hospitable.

The reception at Oakland, and the excursion around the bay, were characterized by a degree of order, harmony, and social enjoyment, not excelled in any particular by similar occasions in the other cities the Association has visited.

As we have already said, the general sessions were devoted to the regular order of business, in the transaction of which there was maintained good order, uniform courtesy, and more freedom of debate than in most of the previous meetings. It is true that a few words of a personal character passed between Dr. W. L. Atlee and Dr. H. R. Storer, for which they both apologized before they sat down. So, an unfortunate remark in relation to the drinking habits of a portion of the profession, by Dr. H.

Gibbons, Sen., in the last evening session, called out a moderate hissing from a part of the assembly, but which was satisfactorily explained before he left the platform. Can our criticising friends point to an assembly of three or four hundred members of the legal, clerical, political, agricultural, or any other profession, which has transacted business in general sessions four successive days without exhibiting more discord, more personal allusions, and less rigid attention to business than was seen at the meeting in SanFrancisco? If so, they have been more fortunate in their observations than we have. The most marked fault in the meeting at SanFrancisco arose from a defect in the arrangements for the sections. When the first morning session closed, and the various reports and papers had been referred to the several sections, there was no notice where the sections were to meet, and no member of the Committee of Arrangements present to give any information. The consequence was that only one section met that afternoon, and it was not until the afternoon of the second or third days that the other sections organized, and even then, that on Practical Medicine and Obstetrics occupied a small dark room that very few found. With this exception, the work of the Committee of Arrangements was well done, and the profession of SanFrancisco deserve much credit.

From a pretty careful observation of the doings of our national organization, we are satisfied that nine-tenths of all the waste of time, unpleasant discussions, and actions of questionable propriety, that take place from year to year, arises from the persistent disposition of some members to make use of the Association to serve their own personal ends. One is afraid that some medical college in his neghborhood will put its fees lower than the one with which he is connected, and straightway he comes up to the Association armed with a resolution solemnly declaring all attempts to cheapen medical education derogatory to the *dignity* and honor of the profession. Another finds that he has been underbidden by some brother doctor, for the contract to attend the county poor-house, and he comes with a resolution declaring it highly unprofessional for any member of the profession to render *contract* service, either to individuals or public institutions. Another becomes envious of the temporary success of some advertising mountebank, and he comes with a resolution to amend the Code of Ethics. Still another finds among the regulations of his local medical society some rule or by-law that conflicts with his notions of propriety, and he comes up to the national gathering fully determined to get some action adopted which will practically nullify the objectionable rule, and enable him to go home and set the local society at defiance. In this way topics are forced upon the attention of the Association, discussions elicited, and time wasted, concerning matters over which it can exercise no legitimate control.

The American Medical Association was organized for the accomplishment of three great and important purposes, namely, the promotion of harmony, social intercourse, and unity of action; the improvement of medical education; and the advancement of medical science and practice. So long as it adheres rigidly to these purposes it will continue to live, prosper, and do good. But just so far as it lends itself to the work of assuaging private griefs, advancing local and individual interests, or intermeddling with matters that belong exclusively to individuals or local societies, it will lose the confidence and respect of the intelligent and honorable part of the profession everywhere.

---

THE twentieth annual meeting of the American Association for the Advancement of Science will be held at Indianapolis, Ind., commencing August 16th, 1871. A pleasant and profitable meeting is expected.

---

THE PHYSIOLOGICAL EFFECTS OF SEVERE AND PROTRACTED MUSCULAR EXERCISE; WITH SPECIAL REFERENCE TO ITS INFLUENCE UPON THE EXCRETION OF NITROGEN.—During the month of November, 1870, the celebrated pedestrian, Edward Payson Weston, made an attempt to walk four hundred miles in five consecutive days. In May previous, Mr. Weston accomplished the extraordinary feat of walking one hundred miles in twenty-one hours, thirty-nine minutes. Prof.

Austin Flint, Jr., had an opportunity to examine the entire urine passed by Mr. Weston during this walk. The results of these examinations were noticed in the EXAMINER at the time of their publication by Prof. Flint. Unfortunately, however, these investigations were based upon very imperfect and unsatisfactory data; when, therefore, Mr. Weston, previous to his attempted four hundred mile walk, kindly offered to submit himself to any scientific observations that Prof. Flint might desire to undertake in connection with the effort, the proposition was of course gladly accepted. Prof. A. Flint, Jr., with the co-operation of Profs. J. C. Dalton, Alex. B. Mott, W. H. Van Buren, Austin Flint, and W. A. Hammond, undertook a very careful, thorough, and complete series of observations, extending over five days previous to the walk, the five days during the walk, and the five days immediately succeeding to it. A full account of these observations, with the conclusions drawn from them, is published by Prof. A. Flint, Jr., in the New York *Medical Journal* for June, 1871. A reprint of the article from that journal has since been issued in book form by Messrs. D. Appleton & Co., New York.

The object aimed to be accomplished by the experiments was to throw light upon some obscure and unsettled points connected with the subject of nutrition and disassimilation, and especially to determine, if possible, the influence of muscular exercise upon the excretion of nitrogen. As introductory to the subject, Prof. Flint gives a brief resumé of the generally received physiological views regarding certain of the points involved. The well-known theory of Leibig, which claims the elimination of nitrogen to be, to a great extent, a measure of the waste of the nitrogenized elements of the tissues, and that this is increased by exercise, was for some time accepted by physiologists almost without question. This theory was, however, modified a few years later by the researches of Lehman, who showed, by a large number of observations on his own person, that, other conditions being equal, the character and quantity of food modified very greatly the elimination of urea. The views of Leibig, as modified by the researches of Lehman, were pretty generally accepted up to 1866, when Fick and Wislicenus published an account of experiments made in ascending one of the Alpine peaks. From their experiments they concluded that muscular exercise does not necessarily increase the elimination of nitrogen; that the substance of muscle itself is consumed in insignificant quantity; and that the muscular system is a machine, consuming, in its work, not its own substance, but fuel, which is supplied by the food. The most efficient fuel they considered to be non-nitrogenized food; the results of its consumption being force, or work, heat and carbolic acid. These theories and experiments have changed very materially the current of physiological opinion with regard to the origin of muscular force and the significance of the elimination of nitrogen.

The following is a brief outline of the course followed by Prof. Flint in making the observations: 1st. Mr. Weston was placed in the hands of Mr. Thos. C. Doremus, Jr., who did not leave him day or night for the fifteen days, except on two occasions, when he was relieved by Mr. Loew and Prof. Flint. During the walk, Profs. Doremus, Mott, or Flint, Jr., one or all of them were constantly present. 2d. Each day at the same hour, and, as nearly as possible, under the same conditions, the naked weight, pulse, respirations, and temperature were noted. 3d. The weight of every separate article taken as food or drink was recorded. "This was done for two purposes; to note the ingesta and excreta, with reference to the weight of the body; and to have all the articles of food separately weighed, so as to estimate the daily consumption of nitrogen." 4th. "The amount of exercise taken each day, in the first period, before the walk, and in the third period after the walk, and also anything unusual in his general condition was noted."

5th. The entire urine of the twenty-four hours was collected day by day, for the purpose of subjecting it to chemical and microscopical examination. This course was carefully and faithfully carried out, each day's observations being given in full in Prof. Flint's article. The following table, which we copy from the article, contains a brief summary of the results:

TABLE D.

**Daily Averages for the Three Periods.**

(French weights and measures in parentheses.)

| | First Period—Five Days before the Walk. | Second Period—Five Days of the Walk. | Third Period Five Days after the Walk. |
|---|---|---|---|
| Weight | Loss in 5 days—21.8 oz. (593 gr.) | Loss in 5 days—55.2 oz. (1,565 gr.) Loss in 4 days—83.2 oz. (2,358 gr.) | Gain in 5 days—80 oz. (2,268 gr.) |
| | Average of 5 days— | Average of 5 days— | Average of 5 days— |
| Temperature | 99° Fahr. (37.2° C.) | 96.3° Fahr. (35.7° C.) | 98.6° Fahr. (37° C.) |
| Pulse | 78 | 90 | 74 |
| Respirations | 22 | 21 | 23 |
| Sleep | 8 h. 5 min. | 3 h. 17 m. | 8 h. 29 m. |
| Miles walked | 8.2 miles | 63.5 miles | 2.2 miles |
| Ingesta | 100.50 oz. (2,848.82 gr.) | 171.47 oz. (4,860.57 gr.) | 144.59 oz. (4,098.62 gr.) |
| Nitrogen of food | 339.46 grains (21.994) | 234.76 grains (13.211) | 440.93 grains (28.569) |
| Cutaneous and pulmonary exhalation | 61.63 oz. (1,690.91 gr.) | 138.41 oz. (3,875.18 gr.) | 62.82 oz. (1,706,78 gr.) |
| URINE. | | | |
| Quantity | 37.84 fl. oz. (1,134.0 c. c.) | 38.46 fl. oz. (1,138.0 c. c.) | 58.14 fl. oz. (1,720.3 c. c.) |
| pecific gravity | 1024.9 | 1028.7 | 1023.0 |
| Urea | 628.24 grains (40.705) | 722.16 grains (46.803) | 726.79 grains (47.094) |
| Nitrogen in urea | 293.18 " (18.729) | 337.01 " (21.841) | 339.17 " 21.977) |
| Uric acid | 2.26 " 0.127) | 3.00 " (0.194) | 1.42 " (0.082) |
| Phosphoric acid | 50.14 " (3.262) | 76.63 " (4.965) | 56.89 " (3.674) |
| Sulphuric acid | 41.57 " (2.693) | 53.50 " (3.666) | 49.02 " (3.176) |
| Chloride of sodium | 159.45 " (10.331) | 65.08 " (4.217) | 312.40 " (20.241) |
| FÆCES. | | | |
| Quantity | 4.08 oz. (115.6) | 4.53 oz. (128.3) | 6.33 oz. (179.3) |
| Nitrogen | 21.91 grains (1.421) | 24.32 grains (1.576) | 33.99 grains 2.202) |
| Nitrogen in urea and fæces combined | 315.09 grains (20.149) | 361.52 grains (23.217) | 373.15 grains (24.179) |
| Nitrogen of urea and fæces per 100 pts. of Nitrogen of food | 95.53 parts | 174.81 parts | 91.93 parts |
| Uric acid per 100 pts. of urea | 0.362 parts | 0.409 parts. | 0.187 parts |

In regard to the remarkable variation in weight, shown by the table in the three different periods, Prof. Flint says:

"*Causes of the Variations in Weight.**—In a measure, the

* To avoid complicating the discussion of the causes of the variations in weight, the English weights only will be used.

variations in weight during the fifteen days may be satisfactorily explained; but there are certain questions involved that are as yet obscure. The explanation of the variations during the walk, and for the five days after, is much facilitated by a comparison of the ingress and egress of nitrogen.

"At the outset of the investigations, the weight was 120.5 lbs., which Mr. Weston thought was about normal. During the period of five days before the walk, the variations were not very great, the highest being 12 oz. above, and the lowest 32 oz. below. At the end of the fifth day, the weight was reduced by about 21 oz. On the first day, the weight being unchanged, the exercise was fifteen miles. The food was of the ordinary variety, but its quantity and proportion of nitrogen were about 30 per cent. above the average for an ordinary man. On the second day, the diminished exercise, the food being less, but still above the normal average, will account for the increase in weight of 12 oz. On the third day, the exercise was the same as on the second day, but the food was reduced a little below the normal average, which will account for 20 oz. loss of weight. On the fourth day, the food was still below the average, being about the same as on the previous day, but it contained a large proportion of nitrogenized matter, over 20 per cent. more than on the third day. The exercise was fifteen miles, which, with the diet, will account for 24 oz. loss of weight. On the fifth day, the food was increased to a little above the average, and it contained an immense amount of nitrogen, about 35 per cent. above the average. This fact, with the absolute muscular repose and ten hours' sleep, as a preparation for the walk, will readily account for 11 oz. increase in weight. During this period of five days before the walk, the average quantity of food and drink was 100.5 oz., containing 339.46 grains of nitrogen, the ordinary average being 90 oz., containing 310 grains of nitrogen. The average discharge of nitrogen by the urine and fæces was 95.53 parts per 100 parts of the nitrogen of food; which is about normal. It is thus evident that the variations in weight during a period of five days of ordinary life can be readily explained in accordance with generally-accepted physiological principles.

"In endeavoring to explain the variations in weight that occurred during the walk, and for the succeeding five days, the extraordinary amount of muscular exertion introduces new elements to be considered. These have a most important bearing upon the subject of nutrition, disassimilation, and 'the source of muscular power,' about which so much has been written within the past few years.

"First, What tissue was consumed, the products being thrown off, during the effort of walking 317½ miles in five consecutive days? Was it the muscular substance? The importance, as regards our ideas of nutrition, of a positive and definite answer to this question can hardly be overestimated.

"The loss of weight was undoubtedly due in a great measure to the excessive muscular exertion; but in part, also, to change in diet. This proposition does not demand discussion.

"The loss must have been either in liquids, fats, or muscular substance.

"It is not probable that the loss was due, to any great extent, to a diminution in the proportion of liquids, for the excessive loss from the skin was instantly supplied by liquids taken into the stomach. It is not necessary to cite experiments which show that loss by the skin, as it occurs in hot-air or vapor-baths, or in working for an hour or more at a high temperature, is readily compensated by liquid ingesta, as this fact is well settled in physiology.* A glance at the daily tables of food and drink will show that, during the five days of the walk, Mr. Weston took from 8 lbs. 8 oz. to 10 lbs. 11 oz. of liquids.

"If the loss were due to a consumption of non-nitrogenized matters, it would be chiefly of fat and would be represented by the carbonic acid of expiration. It is certain that the non-nitrogenized constituents of the body do not contribute to the formation of the nitrogenized excrementitious matters.

"If the loss were due to a consumption of the nitrogenized elements of the body, principally of the muscular tissue, this loss, under the extraordinary muscular effort, would be represented by the nitrogen of the excretions. It is not probable that the nitrogenized constituents of the body are, in any considerable amount, changed into non-nitrogenized matter, and exhaled under the form of carbonic acid, though this may occur to a slight extent.

"The question then resolves itself to that of the relative consumption and elimination of nitrogenized matters. The following are the facts on this point, observed during the five days of the walk:

"During the five days of the walk† Mr. Weston consumed in all, 1,173.80 grains (76.055 grammes) of nitrogen in his food. During the same period, he eliminated 1,807.60 grains (116.084 grammes) of nitrogen in the urine and fæces. This leaves 633.80 grains (40.030 grammes) of nitrogen, over and above the nitrogen of the food, which must be attributed to the

* See my work on Physiology, New York, 1870, vol. iii., p. 140, *et seq.*

† I have reduced these calculations, on account of their great importance, to grammes.

waste of his tissues, and probably almost exclusively to the waste of his muscular tissue. According to the best authorities, lean meat, uncooked, or muscular tissue, contains 3 per cent. of nitrogen.* The loss of 633.80 grains (40.030 grammes) of nitrogen, would then present a loss of 21,127.00 grains (1,334.33 grammes), or 3.018 lbs. of muscular tissue. The actual loss of weight was 3.450 lbs. (1,565.00 grammes). This allows about 0.43 lb. (230.67 grammes) loss unaccounted for, which might be fat or water.

"The correspondence of these figures of loss calculated from the amount of nitrogen eliminated with the actual loss in weight leaves no room for doubt with regard to the fact that the immense exertion during this period of five days was attended with consumption of the muscular substance. Those who have adopted the view that the muscular system is like a steam-engine, consuming in its work food as fuel and not its own substance, may say that this is an extraordinary case, as it undoubtedly is; but the facts developed by the foregoing observations prove, none the less conclusively, that the muscular system may consume its own substance by exercise, even when the individual takes all the food required by his appetite. It can hardly be, however, that the foregoing facts are not in accordance with a general physiological law.

"It will be interesting, now, to study the behavior of the system after the walk, when there was almost absolute repose, and when the quantity of nitrogen taken with the food was largely increased. The important question here is the following:

"In the return of the weight to the normal standard, did the muscular tissue take up nitrogen to repair the excessive waste engendered by the five days of exertion?

"In two days after the walk, the weight had increased to within four ounces of the standard at the beginning of the observations, five days before the walk. It is not to be expected that this increase would be due entirely to appropriation of nitrogenized matter by the muscular system. Reference to the tables of diet for these two days shows that the food taken was about 155 oz. each day, the normal average being assumed at 90 oz., an excess of a little more than 70 per cent. The nitrogen taken was about 50 per cent. in excess of the normal amount. The tables also show a large proportion of non-nitrogenized matter in the food on those days. The exercise was only two miles daily. Mr. Weston gained in weight 4.5 lbs. He retained in his system an amount of nitrogen equivalent to 1.1 lb.

* Payen, *Precis theorique et pratique des substances alimentaires*, Paris, 1865, p. 488.

In view of the muscular inactivity and the large proportion of non-nitrogenized matter in the food, it is fair to assume that the remaining 3.4 lbs. was due to accumulation of fat. This, however, is a point incapable of positive demonstration. Taking the entire period of five days after the walk, the gain in weight was five pounds, which brought it 4 oz. above the weight at the beginning of the fifteen days. The excess of the nitrogen of food over the nitrogen of the urine and fæces represented, for these five days, an accumulation of 1.6 lb. of muscular substance. During this time there was almost complete repose of the muscular system. The daily quantity of food was about 61 per cent. over the normal average, and the nitrogen, about 42 per cent. over the average. The food contained, also, a large proportion of non-nitrogenized matter.

"These facts seem to indicate that, after the immense effort in walking 317½ miles in five consecutive days, for five days of muscular inactivity, the quantity of food being large and containing a greater proportion of non-nitrogenized matter than the food taken either before or during the walk, the muscular system appropriated 1.6 lb. of nitrogenized matter, and the entire body accumulated about 3.4 lbs. of fat. It is well known that athletes, after a season of severe training by exercise and nitrogenized diet, accumulate fat very rapidly, when the muscles are allowed repose and the diet is unrestricted."

"The following conclusions were arrived at in regard to the influence of nitrogenized food upon the excretion of nitrogen:

"Every day that an excess of nitrogenized food was taken, it was followed, on the succeeding day, and, on one occasion, on the succeeding two days, by a largely increased discharge of nitrogen in the urea and fæces, the discharge on these days exceeding the amount taken in the food; but the general average for five days, during the period of five days before the walk, and the period of five days after the walk, was from 92 to 95 parts of nitrogen discharged, for every 100 parts of nitrogen introduced.

"The average for the five days after the walk shows an introduction of 440.93 grains (28.569 grammes) of nitrogen daily, an excess of about 42 per cent. over the average for an ordinary man. For every 100 parts of nitrogen in the food, the average daily excretion, during this period, was 91.93 parts."

"As regards the elimination of phosphoric and sulphuric acid, the following averages were obtained:

"For the first period, five days before the walk, the average discharge of phosphoric acid was 50.14 grains (3.262 grammes), and of sulphuric acid, 41.57 grains (2.693 grammes). The average quantity of nitrogen in the food was 339.46 grains (21.994 grammes).

"For the second period, five days of the walk, the average discharge of phosphoric acid was 76.63 grains (4.965 grammes), and of sulphuric acid, 53.50 grains (3.666 grammes). The average quantity of nitrogen in the food was 234.76 grains (13.211 grammes).

"For the third period, five days after the walk, the average discharge of phosphoric acid was 56.89 grains (3.674 grammes), and of sulphuric acid, 49.02 grains (3.176 grammes). The average quantity of nitrogen in the food was 440.93 grains (28.569 grammes).

"These averages show that the walk of 317½ miles in five consecutive days increased the excretion of phosphoric acid more than 50 per cent. over the excretion under ordinary conditions, notwithstanding that the nitrogenized food was diminished 31 per cent. Under the same conditions, there was an increase of about 30 per cent. in the excretion of sulphuric acid. The influence of exercise upon the excretion of the phosphates and sulphates, irrespective of the composition of the food, cannot be doubted.

"A comparison of the averages for the first period, five days before the walk, and the third period, five days after the walk, shows an increase in the excretion of phosphoric acid, for the third period, of 13.4 per cent., with an increase of 30 per cent. in the quantity of nitrogenized food. Under the same conditions, the excretion of sulphuric acid was increased 19.2 per cent."

"The average quantity of urine passed during the five days was 38.46 fl℥. The average quantity of liquids taken was 150.40 fl℥. This average of 38.46 fl℥ against 38.14 fl℥, the average for the five days previous to the walk shows conclusively that the walk of 316½ miles in five days did not affect the quantity of urine; and that the immense amount of liquids taken during that time must have been discharged by the skin."

Prof. Flint says in conclusion:

"It is evident, from the results of these investigations, that the great question of the influence of muscular exercise upon the elimination of nitrogen can be accurately studied only by comparing the nitrogen of the food with that of the excretions; and this should be done, if possible, upon a perfectly physiological diet. It is indispensable, also, to extend the experiments over periods of several days each; otherwise the results will necessarily be confused and unsatisfactory. The observations with regard to the weight and various other conditions were necessary to control the more important points to be considered."

### Mortality for the Month of June, 1871.

| | |
|---|---|
| Accidents, crushed | 1 |
| " asphyxia | 1 |
| " drowned | 6 |
| " gun-shot wound | 2 |
| " fracture of skull | 1 |
| " run over by wagon | 1 |
| " by railroad cars | 3 |
| " scalded | 1 |
| Abcesses, general | 1 |
| Abortion | 1 |
| Anæmia | 1 |
| Apoplexy | 7 |
| Births, premature | 26 |
| " still | 48 |
| Bowels, obstruction of | 1 |
| Brain, congestion of | 7 |
| " inflammation of | 14 |
| " softening of | 1 |
| Bronchitis | 8 |
| " capillary | 4 |
| Cancer of breast | 2 |
| " of groin | 1 |
| " of kidney | 1 |
| " of stomach | 1 |
| " of uterus | 2 |
| Childbirth | 1 |
| Cholera infantum | 67 |
| " morbus | 1 |
| Consumption | 43 |
| Convulsions | 67 |
| " puerperal | 1 |
| Croup | 4 |
| " diphtheritic | 1 |
| " membraneous | 1 |
| Cyanosis | 4 |
| Cynanche trachealis | 1 |
| Cystitis, chronic | 1 |
| " acute | 1 |
| Debility, general | 4 |
| Development deficient | 1 |
| Diarrhœa | 19 |
| " and complications | 3 |
| Diphtheria | 6 |
| Dropsy, general | 4 |
| " of peritonæum | 1 |
| Dysentery | 17 |
| " chronic | 1 |
| Entero colitis | 5 |
| Enteritis | 9 |
| Epilepsy | 1 |
| Erysipelas | 3 |
| Fever, puerperal | 3 |
| " scarlet | 2 |
| " " & complicat's | 1 |
| " typhoid | 4 |
| Gastritis | 2 |
| Hæmorrhagica purpuria | 1 |
| " internal | 1 |
| " umbilical | 1 |
| Heart disease | 1 |
| " atrophy of | 1 |
| " fatty degeneration of | 1 |
| " valvular disease of | 3 |
| Hip-disease | 1 |
| Hip-joint, injury of | 1 |
| Hemiplegia | 1 |
| Hernia, strangulated | 1 |
| Hydrocephalus | 10 |
| " acute | 5 |
| " chronic | 3 |
| Inanition | 10 |
| Intemperance | 1 |
| Icterus | 1 |
| Kidneys, Bright's disease of | 1 |
| Liver, abscess of | 1 |
| " hypertrophy of | 1 |
| " inflammation of | 2 |
| Lead colic | 1 |
| Lungs, congestion of | 5 |
| " and liver, congestion of | 1 |
| " hemorrhage of | 2 |
| Manslaughter | 1 |
| Malformation | 1 |
| Measles | 19 |
| " and complications | 20 |
| Metroperitonitis | 1 |
| Meningitis | 9 |
| " and peritonitis | 1 |
| " cerebro-spinal | 4 |
| " tubercular | 2 |
| Metritis and phlebitis uterine | 1 |
| Myelitis | 1 |
| Neuralgia | 1 |
| Old age | 5 |
| Œdema pulmonum | 1 |
| Paralysis of abdominal viscera | 1 |
| Paralysis | 3 |
| " and congestion of brain | 1 |
| Peritonitis | 1 |
| " puerperal | 1 |
| Pneumonia | 22 |
| " typhoid | 4 |
| Purpura, acute | 1 |
| Pyæmia | 1 |
| Rheumatism | 1 |
| " inflammatory | 1 |
| Scrofula | 3 |
| Small-pox | 3 |
| Spine, tuberculosis of | 1 |
| Sunstroke | 1 |
| Suicide by shooting | 1 |
| " by hanging | 2 |
| " by morphia | 1 |
| Tabes mesenterica | 19 |
| Teething | 6 |
| Tetanus traumatic | 2 |
| Uterus, hemorrhage of | 1 |
| Vertebra, fracture of | 1 |
| Whooping-cough | 6 |
| " and complications | 2 |
| Unknown | 1 |
| Total | 558 |

### COMPARISON.

Deaths in June, 1871, - 558 | Deaths in June, 1870, -- 720 | Decrease, 162

Deaths in May, 1871, 525 | Increase, 33

### AGES.

| | |
|---|---|
| Under 1 | 248 |
| 1 to 2 | 65 |
| 2 to 3 | 24 |
| 3 to 4 | 21 |
| 4 to 5 | 8 |
| 5 to 10 | 19 |
| 10 to 20 | 26 |
| 20 to 30 | 41 |
| 30 to 40 | 32 |
| 40 to 50 | 30 |
| 50 to 60 | 13 |
| 60 to 70 | 17 |
| 70 to 80 | 9 |
| 80 to 90 | 4 |
| 90 to 100 | — |
| 100 to 101 | — |
| Unknown | 1 |
| Total, | 558 |

| | | | | | |
|---|---|---|---|---|---|
| Males | 316 | Single | 453 | White | 549 |
| Females | 242 | Married | 105 | Colored | 9 |
| Total | 558 | Total | 558 | Total | 558 |

NATIVITY.

| | | | | | |
|---|---|---|---|---|---|
| Austria | 1 | France | 1 | Poland | 1 |
| Bohemia | 4 | Germany | 42 | Russia | 1 |
| Canada | 8 | Holland | 2 | Scotland | 4 |
| Chicago, Native | 86 | Ireland | 49 | Switzerland | 2 |
| Chicago, Foreign | 247 | Italy | 3 | Sweden | 4 |
| U. S., other parts | 76 | Newfoundland | 1 | Unknown | 5 |
| Denmark | 2 | Norway | 9 | | |
| England | 9 | Nova Scotia | 1 | Total | 558 |

MORTALITY BY WARDS FOR THE MONTH.

| Wards. | Mortality. | Pop. in 1870. | One death in |
|---|---|---|---|
| 1 | 7 | 6,531 | 933 |
| 2 | 12 | 14,338 | 1195 |
| 3 | 27 | 16,805 | 622 |
| 4 | 14 | 12,178 | 870 |
| 5 | 13 | 11,605 | 893 |
| 6 | 45 | 19,486 | 443 |
| 7 | 32 | 13,849 | 433 |
| 8 | 58 | 22,994 | 396 |
| 9 | 60 | 27,278 | 455 |
| 10 | 17 | 13,750 | 809 |
| 11 | 24 | 14,988 | 620 |
| 12 | 21 | 13,976 | 665 |
| 13 | 4 | 8,943 | 2236 |
| 14 | 9 | 9,076 | 1009 |
| 15 | 44 | 20,382 | 463 |
| 16 | 18 | 13,975 | 776 |
| 17 | 26 | 17,118 | 658 |
| 18 | 35 | 17,069 | 488 |
| 19 | 4 | 8,738 | 2184 |
| 20 | 20 | 13,628 | 681 |

| Mortality. | |
|---|---|
| Accidents | 16 |
| County Hospital | 11 |
| Foundling Home | 15 |
| Home for Friendless | 3 |
| Hospital Alexian Brothers | 1 |
| Hospital for Women and Children | 1 |
| Immigrants | 6 |
| Jewish Hospital | 1 |
| Marine Hospital | 1 |
| Mercy Hospital | 5 |
| Manslaughter | 1 |
| Protestant Orphan Asylum | 1 |
| St. Luke's Hospital | 1 |
| St. Joseph Orphan Asylum | 1 |
| Suicide | 4 |
| Total | 558 |

Mean Thermometer for month, 72°; Rain, 5.940 inches; Deaths daily, 18½.

Compared with the month of May, there is an increase of only 38 in the number of deaths, indicating no unusual condition of the public health, although there is considerable difference in the cause of the same. There were 7 more deaths by brain diseases, 62 by cholera infantum, 19 by convulsions 14 by diarrhœa, 10 by dysentery, 5 by meningitis, 8 by tabes mesenterica, and 2 by whooping cough; while there were 14 less by accidents, 5 by bronchitis, 8 by consumption, 8 by croup, 4 by diphtheria, 3 by dropsy, 8 by scarlet fever, 6 by typhoid fever, 17 by heart diseases, 3 by inanition, 6 by old age, 17 by pneumonia, and 3 by teething. Adult mortality diminished, while infantile mortality increased.

The decrease compared with the corresponding month of 1870 is very great. There were 5 more deaths by bronchitis, 2 by erysipelas, 2 by congestion of the lungs, 22 by measles, 14 by pneumonia, and 8 by whooping cough, while there were 5 less by accidents, 2 by cancer, 2 by cholera morbus, 78 by cholera infantum, 2 by consumption, 22 by convulsions, 10 by croup, 26 by scarlet fever, 6 by typhoid fever, 3 by heart diseases, 2 by laryngitis, 2 by meningitis, 2 by old age, 2 by small-pox, 7 by sunstroke, and 13 by teething. It will be seen that certain infantile diseases have diminished the death-rate, while others increased it, though there is a general decrease in consequence of atmospheric influences. The mean temperature of the month is the same, but there is a difference in the range of 16°, which will explain the decrease in the number of deaths, and this great change was incident to the last week of the month last year, which was the hottest of the year.

There is, this year, observable what might be termed a more decided hemorrhagic tendency than usual, although, as I remarked before, the death-rate is much lower than last year, in consequence of the most powerful climatic influences. The following table will show the meteorological conditions and number of deaths since 1866, for the month of June, plainly showing their influence, independent of any contagions of epidemic influence:

| June. | 1866. | 1867. | 1868. | 1869. | 1870. | 1871. |
|---|---|---|---|---|---|---|
| Highest temperature | 99° | 84° | 88° | 83° | 100° | 88° |
| Lowest " | 50° | 46° | 50° | 49° | 54° | 58° |
| Range of " | 40° | 38° | 38° | 34° | 46° | 40° |
| Mean " | 70 | 72.4 | 66.3 | 65.4 | 72 | 72 |
| Rain | 2.50 | 1.86 | 3.10 | 5.03 | 1.70 | 5.94 |
| Mortality | 319 | 283 | 305 | 434 | 720 | 558 |

It will be seen that the death-rate was highest for this month in the 8th ward, next the 6th and 7th wards, the number of deaths in proportion to the population being the same; then in the 19th, 15th, 18th, 11th, 3rd, 17th, 12th, 20th, 16th, 10th, 4th, 5th, 1st, 14th, 2nd, 9th, and lowest in the 13th ward. In addition to all local causes, it will be observed that there is an increase in the number of deaths in the wards contagious to the Chicago River, particularly in the south-western part of the city, plainly demonstrating the effect of the exhalation of the Chicago River and the north-east wind upon life during this month.

JOHN H. RAUCH, *Sanitary Superintendent.*

---

SPINA BIFIDA.—Dr. Dyes, in the *Deutsche Klinik* of 18th March, relates how in his younger days a postilion came to him complaining of a swelling in the region of the sacrum, which lay a little above the os coccygis, and prevented him from sitting. Thinking that this was an abscess, the doctor made a small incision, and some clear fluid issued forth. In about five minutes the patient began to shiver, convulsions supervened, and in ten minutes he was a corpse. The swelling was spina bifida.—*The Doctor.*

---

MONEY RECEIPTS FROM MAY 20 TO JUNE 20.—Dr. J. S. Sherman, $6.00; Dr. J. C. Corbus, 5.00; Dr. J. D. Mitchell, 6.00; Dr. M. Davis, 5.00; Dr. D. T. Martyn, 3.00; Dr. M. Risch, 3.00; Dr. J. B. Meigs, 2.00; Dr. J. N. Lilly, 3.00; Dr. J. Priestmon, 3.00; Dr. J. R. Webster, 3.00; Dr. Quoles, 6.00; Dr. Quirck, Drs. Peck and Moore, 3.00; Dr. J. B. Newman, 3.00; Dr. J. N. Barlow, 3.00; Dr. L. Tibbetts, 3.00; Dr. J. J. Hale, 1,50; Dr. J. Hurley, 3,00; Dr. O. Given, 9,00; Dr. W. L. Pollock, 3,00; Dr. J. M. Woodworth, 4,50; Dr. O. S. Moxon, 3,00.

